# Translation, cross-cultural adaptation, and validity of the Brazilian version of the Cognitive Function Instrument

**DOI:** 10.1590/1980-5764-DN-2021-0057

**Published:** 2022

**Authors:** Adalberto Studart-Neto, Natália Cristina Moraes, Raphael Ribeiro Spera, Silvia Stahl Merlin, Jacy Bezerra Parmera, Omar Jaluul, Mônica SanchesYassuda, Sonia Maria Dozzi Brucki, Ricardo Nitrini

**Affiliations:** 1 Universidade de São Paulo, Hospital das Clínicas, Faculdade de Medicina, Departamento de Neurologia, São Paulo SP, Brazil.; 2 Universidade de São Paulo, Hospital das Clínicas, Faculdade de Medicina, Departamento de Geriatria, São Paulo SP, Brazil.; 3 Universidade de São Paulo, Escola de Artes, Ciências e Humanidades, Programa de Pós-Graduação em Gerontologia, São Paulo SP, Brazil.

**Keywords:** Cognitive Dysfunction, Adaptation, Validation Study, Cognition, Disfunção Cognitiva, Adaptação, Validation Study, Cognição

## Abstract

Subjective cognitive decline (SCD) is defined as a self-perception of a progressive cognitive impairment, which is not detected objectively through neuropsychological tests. The Alzheimer's Disease Cooperative Study developed the Cognitive Function Instrument (CFI) to evaluate individuals with SCD. The CFI consists of two versions, namely, a self-report and a partner report.

**Objective:**

This study aimed to translate CFI into Brazilian Portuguese, perform a cross-cultural adaptation, and validate the Brazilian version.

**Methods:**

The translation and transcultural adaptation process consisted of six stages, and the preliminary version was answered by a sample of individuals recruited among the patients’ caregivers from a cognitive neurology outpatient clinic. Finally, the final Brazilian version of the CFI was applied to a sample of nondemented older adults to validate the instrument, which was divided into with and without SCD, according to the answer “*yes*” for the question: “*Do you feel like your memory is becoming worse?*”.

**Results:**

The final version of CFI showed a high level of acceptability as an assessment tool in nondemented older adults. Participants with SCD had higher scores in the CFI self-report compared with those without complaints. In the receiver operating characteristic curve analysis, the area under the curve of the CFI self-report was 0.865 (95% confidence interval 0.779–0.951), and the cutoff score of 2.0 was the one that best distinguished the SCD group from the control group, with a sensitivity of 73.3% and a specificity of 81.5%.

**Conclusions:**

CFI proved to be an instrument with good accuracy and easy applicability to identify older adults with SCD.

## RESUMO.

O declínio cognitivo subjetivo (DCS) é definido como uma autopercepção de um comprometimento cognitivo progressivo, não detectado objetivamente por meio de testes neuropsicológicos. O *Alzheimer's Disease Cooperative Study* desenvolveu o instrumento de função cognitiva (IFC) para avaliar indivíduos com DCS. O IFC existe em duas versões, uma do paciente e outra do acompanhante.

**Objetivo:**

O objetivo deste estudo foi traduzir para o português brasileiro, fazer uma adaptação transcultural e validar a versão brasileira do IFC.

**Métodos:**

O processo de tradução e adaptação transcultural consistiu em seis etapas, e a versão preliminar foi respondida por uma amostra de voluntários recrutados entre os cuidadores de pacientes de um ambulatório de Neurologia Cognitiva. Por fim, a versão brasileira final do IFC foi aplicada a idosos sem demência, que foram divididos naqueles com e sem DCS de acordo com a resposta “sim” à questão: “Você sente que a sua memória está piorando?”.

**Resultados:**

A versão final do IFC mostrou alto nível de aceitabilidade como ferramenta de avaliação em idosos sem demência. Os participantes com DCS tiveram pontuações mais altas na versão do paciente em comparação com aqueles sem queixas. Nas análises da curva característica de operação do receptor (ROC), a área sobre a curva da versão do paciente foi de 0,865 (intervalo de confiança [IC95%] 0,779–0,951) e a pontuação de corte de 2,0 foi a que melhor distinguiu o grupo com DCS dos controles, com sensibilidade de 73,3% e especificidade de 81,5%.

**Conclusões:**

O IFC mostrou-se um instrumento de boa acurácia e de fácil aplicabilidade para identificar idosos com DCS.

## INTRODUCTION

Subjective cognitive decline (SCD) is defined as a self-perception of a progressive cognitive impairment, which is not detected objectively through neuropsychological tests^1,2^. Longitudinal epidemiological studies have linked SCD with a higher risk factor for progression to mild cognitive impairment (MCI) and dementia^3–6^. In a meta-analysis of 29 studies, the annual conversion rate from SCD to MCI and dementia was approximately 6.7 and 2.3%, respectively, compared with the conversion rate of only 1% to dementia among older adults without SCD. Moreover, among those studies with a follow-up of more than 4 years, the progression to MCI and dementia reached 26.7 and 14.1%, respectively^7^. Also, the group of older adults with subjective complaints has a higher prevalence of positive biomarkers for Alzheimer's disease (AD)^8–11^.

In 2014, an international working group proposed a diagnostic criterion for SCD, focusing on the standardization of the terminology for research in preclinical AD^12^. However, one of the difficulties pointed out by this task force was the lack of a gold standard instrument for assessment and quantification of SCD. The significant variability and heterogeneity of tools for SCD evaluation have made it difficult to compare research in this area. Some studies have used semi-structured questionnaires, while others have used simple questions like “is your memory getting worse?”. In a nonsystematic review, 19 studies were compared with SCD measures, and 34 self-report questionnaires were identified^13^. Those instruments differed mainly in the mode of administration (by self-administered or interview), in the timeframe by items (while some of them compared the present cognition with weeks or months ago, others compared it with years ago or even younger), and in the assessed cognitive domains (some focused on memory complaints, while others evaluated the decline in other cognitive skills)^13^.

In Brazil, there are few SCD questionnaires translated and adapted to our language and culture. In a systematic review, 25 Brazilian studies were identified, of which 19 used some type of standardized questionnaire^14^. In that review, four types of questionnaires were used in studies and only two had been properly validated.

The Cognitive Function Instrument (CFI) was developed by the Alzheimer's Disease Cooperative Study (ADCS) for the evaluation of subjective cognitive complaints in a group of older adults without dementia^15^. Initially, it was designed to be applied by email and called Mail-In Cognitive Function Screening Instrument (MCFSI); however, it was also used for a self-report on-site assessment. In a longitudinal study with participants of ADCS, CFI was associated with cognitive decline in 48 months^16^. The CFI consists of two versions, i.e., a self-report and a partner report, with 14 questions each. The patient and his/her companion must read and answer the questionnaire independently, without consulting anyone. The items ask about cognitive (i.e., memory, language, and orientation) and functional difficulties, and the issues address the present complaints compared with 1 year ago. Possible responses were “yes” (1), “no” (0), or “maybe” (0.5). The range of scores was 0–14. In some questions (such as driving and performance at work), it is possible to answer “does not apply.”

Therefore, this study aimed to translate the CFI to Brazilian Portuguese, perform a cross-cultural adaptation, and analyze its diagnostic accuracy to identify SCD.

## METHODS

### Development of the Brazilian version of Cognitive Function Instrument

The translation was performed based on the methods proposed by Beaton et al.^17^ During the process of translation and adaptation, the following aspects were observed: semantic equivalence (correspondence in the meaning of words, vocabulary, and grammar), idiomatic equivalence (idiomatic and colloquial expressions should be congruent in the culture in which they were translated), experimental or cultural equivalence (described situations in the original version should be consistent with the cultural context), and conceptual equivalence (maintenance of the concept proposed in the original instrument). In addition, a concern of the translators was to consider the heterogeneity of Brazilian population schooling.

**Figure 1:**
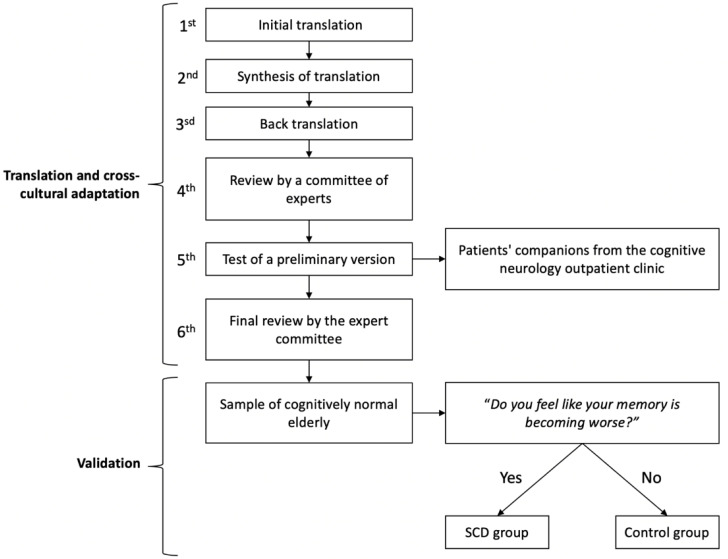
Study design of translation, cross-cultural adaptation, and validity of the Brazilian version of the Cognitive Function Instrument. Preliminary version was applied in a sample of cognitively normal adults recruited among the patients’ companions from the cognitive neurology outpatient clinic of the Hospital das Clínicas of the Medical School of the University of São Paulo. All participants were asked to comment on the instrument, whether they understood each question, what their doubts were, and whether they had any suggestions. After the final Brazilian version, the Cognitive Function Instrument was applied to a sample of cognitively normal elderly to validate the instrument. They were divided into two groups: with subjective cognitive decline (SCD group) and without decline (control group). The participants were recruited from different centers in the city of São Paulo, Brazil.

This transcultural translation and adaptation process consisted of six stages, described below (Figure 1):

- Initial translation: Two translations of the original instrument were prepared independently by two bilingual specialists in the area of cognitive neurology or neuropsychology.
- Synthesis of translation: The two translators met to draw up a synthesis of the two translated versions, and a third member of the project resolved divergences.
- Back translation (or reverse translation): The synthesis of the translation obtained was back-translated into English by two other translators who were also bilingual and specialists in the field. The reverse translations were also developed independently and blind to the original version of the instrument.
- Review by a committee of experts: At this stage, a specialist committee, composed of two other members who did not participate in the translations, met to review all the available versions. The original instrument, the synthesis of the Portuguese translation, and the backward versions were compared. This comparison evaluated the semantic, idiomatic, experimental or cultural, and conceptual equivalences of the terms used in the different versions. From this analysis, a preliminary version of the instrument was translated, adapted, and drafted.
- Test of a preliminary version: After elaborating the preliminary version, it was applied in a sample of cognitively normal adults. All participants were asked to comment on the instrument whether they understood each question, what their doubts were, and whether they had any suggestions.
- Final review by the expert committee: In this last stage, the expert committee again met to prepare the final version. From the comments and suggestions, any changes were applied to obtain the final version. The final Brazilian version of the CFI was used in a group of older adult volunteers without dementia, so that the clinical validity of the instrument could be analyzed.

### Participants

The preliminary version was answered by a sample of individuals recruited among the patients’ relatives from the cognitive neurology outpatient clinic of the Hospital das Clínicas of the Faculdade de Medicina of the Universidade de São Paulo. The inclusion criteria were: 1) to be above 45 years old, 2) to read and write, and 3) to read and understand a text in Portuguese, and 4) to have a next of kin.

The final version was applied to a sample of cognitively unimpaired older adults. They were divided into two groups: with subjective memory complaint (SCD group) and without complaint (control group). The SCD group was defined by the answer “*yes*” for the question: “*Do you feel like your memory is becoming worse?”.* If the participant answered “*no*,” he/she was included in the control group. The participants were recruited from different centers in the city of São Paulo, Brazil: outpatient clinic of the Geriatrics Department of a university hospital (Hospital das Clínicas from the of the Faculdade de Medicina of the Universidade de São Paulo); Open University Program for Senior Citizens at the School of Arts, Sciences and Humanities from the Universidade de São Paulo; and a Senior Center for the Promotion of Healthy Aging. We also recruited community-dwelling older adults through social media and newspapers.

For the sample who completed the final version, the inclusion criteria were: 1) age ≥60 years; 2) schooling ≥4 years; 3) mini-mental state examination (MMSE) normal for education according to the scores obtained by Brucki et al.^18^; 4) clinical dementia rating (CDR) score equal to zero; and 5) 15-question version of the geriatric depression scale (GDS-15)≤5.

The exclusion criteria were: 1) diagnosis of dementia or MCI according to the criteria of the National Institute on Aging and Alzheimer's Association^19,20^; 2) diagnosis of a major psychiatric disorder according to the Diagnostic and Statistical Manual of Mental Disorders, Fifth Edition; 3) history of alcohol or psychoactive drug abuse; 4) current or previous diagnosis of the central nervous system diseases (e.g., stroke or seizure); and 5) both visual and/or auditory limitations that could impair the performance in cognitive tests.

### Assessment procedure

The first assessment was carried out by a neurologist with training in cognitive neurology and consisted of a semi-structured interview with a collection of sociodemographic data; cognitive assessment with MMSE, Montreal Cognitive Assessment (MoCA), and the Brief Cognitive Screening Battery (BCSB)^21,22^ screening for depression and anxiety symptoms using GDS-15 and the Geriatric Anxiety Inventory (GAI), respectively; and the functional assessment with the Functional Activities Questionnaire (FAQ) and the CDR.

In this first assessment, the participant was also asked to read and answer the CFI self-report. The participant was allowed to ask questions to the examiner in case of doubts. When the older adult was unaccompanied, the CFI partner report was given to him, so that a family member could answer and then send it by email.

After the first assessment, the participants who met the inclusion criteria were invited to complete the neuropsychological tests. The tests performed were Forward and Backward Digit Span Test, Trail Making A (TMA) and Trail Making B (TMB), Category (animals) and Letter (FAS) Verbal Fluency Tests, Rey-Osterrieth Complex Figure (copy and delay recall), Logical Memory of the Wechsler Memory Scale, Rey Auditory Verbal Learning Test, and 60-item version of the Boston Naming Test. The estimated intelligence quotient (IQ) was measured with the vocabulary and matrices subtests of the Wechsler Adult Intelligence Scale, Third Edition (WAIS-III). Those who performed at least one test with standard deviation (SD) less than or equal to −1.5 from average normative values adjusted for age and education received a diagnosis of MCI and were excluded from the sample.

### Statistical analysis

Categorical variables were expressed as absolute and relative frequencies and compared with Pearson's chi-square test on univariate analysis. Descriptive statistics including mean, SD, median, and interquartile range (IQR) values were generated for continuous numerical variables. Variables were tested for normality with histogram graphs and the Kolmogorov-Smirnov test. All continuous numerical variables assumed a non-normal distribution and were compared with the Mann-Whitney U test.

Cronbach's alpha for CFI self-report and partner report was used to analyze the internal consistency. The construct validity was obtained from Spearman's rank correlation coefficient (rS) of the total score of CFI self-report and partner report and its items with the other assessment instruments and the neuropsychological test results.

Receiver Operating Characteristic (ROC) curve analysis was used to calculate the area under the curve (AUC) with a 95% confidence interval (95%CI) and the cutoff scores for CFI sensitivity and specificity capable of distinguishing older adults with SCD from those without SCD. Positive and negative predictive values of the questionnaire were also calculated.

The analyses were performed using the Statistical Package for the Social Sciences software, version 21.0 (IBM Statistics, Chicago, IL, USA). All tests were performed considering bilateral hypotheses, and the two-tailed p<0.05 were considered statistically significant.

### Standard protocol approvals, registrations, and patient consents

The study was approved by the Ethics and Research Committee of the Hospital das Clínicas from the Medical School of the Universidade de São Paulo under protocol number 62047616.0.0000.0068. Informed consent was obtained from each participant. The study of human subjects was designed and conducted according to the Declaration of Helsinki.

## RESULTS

### Final Brazilian version of Cognitive Function Instrument

Supplementary Tables 1 and 2 show the final version of the CFI. Eventually, a combination of the two translations was used for the synthetic version (items 1, 4, 7, 8, 9, 10, 11, and 13), while in other items, one of the translations was chosen (items 2, 3, 5, 6, 12, and 14).

On some questions, equivalent terms replaced the original words, for example, the term “decline” replaced by “piorou” (became worse) and “diminuiu’ (decreased) in questions 1 and 10, respectively. Conversely, words were deleted without prejudice to the comprehensive question, as in items 2 (“tend”) and 12 (“substantially”). Such adaptations were aimed mainly at less educated individuals. The term “tax form” was adapted as “imposto de renda” (income tax) because it was closer to the Brazilian cultural context. In contrast, some items were translated literally, without substitution, elimination, or addition of words or terms (such as questions 11 and 14). In other items, examples were inserted to make the question clearer (as in question 9). In addition to the questions, we also adapted the instructions for the questionnaire to make it clearer to the less educated individuals. Supplementary Tables 3 and 4 show all steps of translation and adaptation until the final version was reached.

The preliminary version was applied to 37 individuals. The mean and median age were 63.4 (SD=11.3) and 64.5 (IQR=23) years, respectively. The median schooling was 11.0 (IQR=10) years of schooling. Only eight (21.6%) participants had difficulty in understanding any of the questions or making suggestions.

Five questions were changed between the preliminary and final versions (items 1, 4, 9, 10, and 12). In questions 9 and 12, examples were added to make the questions clearer. Furthermore, words were added and replaced in questions 4 and 10, respectively. Three subjects doubted about the “maybe” option, so we wrote in parenthesis “can be.” Two other subjects had difficulty in understanding “does not apply” option, so we wrote in parenthesis “I do not drive a car” (question 7), “I do not deal with money” (question 8), and “I do not do paid work” (question 10), respectively.

### Validation of Final Brazilian version of Cognitive Function Instrument

The final version was submitted to 72 cognitively normal older adults (48 women) for validation. In only 24 subjects, the CFI partner report was obtained. Forty-five subjects answered “yes” to the question “Do you feel like your memory is becoming worse?” and therefore were classified in the SCD group. The acceptability of the instrument was high because no further adjustments were necessary for the final version during the validation stage.

The median age of the control group was higher than that of the SCD group but without statistical significance. Schooling was similar between the two groups. Older adults in the SCD group had more anxiety and depression symptoms. There was no statistically significant difference between the groups in the neuropsychological tests (Table 1). The SCD group had the highest CFI self-version score, as expected. However, the CFI partner version did not differ between groups (Table 1).

**Table 1:** Demographic information and neuropsychological test scores in a sample of cognitively normal elderly with subjective cognitive decline (SCD group) and without subjective cognitive decline (control group) submitted to final Brazilian version of Cognitive Function Instrument validation. Values expressed as median and interquartile range.

	SCD^a^ group (n=45)	Control group (n=27)	
	Median (IQR^b^)	Median (IQR^b^)	p-value^c^
**Age (y)**	73.0 (13.0)	77.0 (16.0)	0.145
**Education (y)**	16.0 (5.0)	16.0 (6.0)	0.583
**CFI** d **self-report version**	3.0 (3.6)	0.5 (2.3)	<0.001
**CFI** d **partner version**	1.0 (2.6)	0.75 (1.1)	0.323
**Geriatric Anxiety Inventory**	3.0 (7.0)	1.0 (4.0)	0.003
**Geriatric Depression Scale**	2.0 (2.0)	1.0 (1.0)	<0.001
**Intelligence quotient**	109.0 (16.5)	109.0 (15.0)	0.749
**Mini-Mental State Examination**	29.0 (1.0)	29.0 (2.0)	0.418
**Montreal Cognitive Assessment**	25.0 (3.0)	25.0 (4.0)	0.445
**BCSB Delay-Recall** e	8.0 (2.0)	8.0 (2.0)	0.181
**Test Clock Drawing**	10.00 (1.0)	10.00 (1.0)	0.595
**Category Verbal Fluency (animals)**	16.0 (9.0)	19.0 (4.0)	0.072
**Letter Verbal Fluency**	36.0 (19.0)	38.0 (14.0)	0.843
**Logical Memory II**	21.0 (10.0)	21.0 (9.0)	0.675
**RAVLT Delay-Recall** f	8.0 (3.0)	8.0 (3.0)	0.173
**Rey-Osterrieth Complex Figure (Copy)**	36.0 (2.0)	36.0 (2.0)	0.321
**Rey–Osterrieth Complex Figure (Delay-Recall)**	13.0 (11.3)	14.5 (10.0)	0.453
**Trail Making A**	45.0 (15.5)	47.0 (11.0)	0.629
**Trail Making B**	95.0 (44.5)	100.0 (52.0)	0.861
**Forward Digit Span**	8.0 (4.0)	8.0 (4.0)	0.659
**Backward Digit Span**	5.0 (2.0)	6.0 (2.0)	0.244
**Boston Naming Test – 60**	56.0 (5.8)	56.0 (5.0)	0.717

a Subject cognitive decline;

b interquartile range;

c Mann-Whitney test;

d Cognitive Function Instrument;

e Brief Cognitive Screening Battery;

f Rey Auditory Verbal Learning Test.

Approximately half of older adults in the SCD group answered “yes or maybe” when questioned about the worsening of memory in the last year (Table 2). Questions 4 (use of written reminders) and 6 (recalling name and words) were the most frequently answered as “yes or maybe” in both groups (Table 2).

**Table 2:** Percentages of positive answers (yes and maybe) for each item of Cognitive Function Instrument, corrected item-total correlation, and internal consistency (Cronbach's alpha) if the item is deleted in a sample of cognitively normal elderly with and without subjective decline cognitive.

Item	Self-report				Partner report			
	Yes and maybe in SCD group (%)	Yes and maybe in control group (%)	Corrected item-total correlation	Cronbach's alpha if the item is deleted	Yes and maybe (%) in SCD group	Yes and maybe in control group (%)	Corrected item-total correlation	Cronbach's alpha if the item is deleted
1. Subjective memory decline a year ago.	48.9	0	0.50	0.79	28.6	0	0.59	0.63
2. Questions repetition	28.9	11.1	0.21	0.81	7.1	30.0	0.23	0.70
3. Misplacing things	44.5	11.1	0.49	0.79	28.6	10.0	0.66	0.62
4. Use of written reminders	68.9	18.5	0.59	0.78	42.8	20.0	0.26	0.66
5. Remember appointments	24.4	3.7	0.53	0.78	0	10.0	0.11	0.70
6. Recalling name and words	84.4	25.9	0.67	0.77	50.0	20.0	0.45	0.66
7. Driving	17.8	7.4	0.41	0.79	7.1	20.0	0.07	0.70
8. Managing money	11.1	3.7	0.52	0.79	14.3	0	0.61	0.66
9. Social activities	17.8	3.7	0.21	0.81	21.4	10.0	0.28	0.70
10. Work performance	15.5	7.4	0.27	0.80	7.1	0	0.40	0.69
11. Following news or plots of books or movies	22.2	11.1	0.37	0.80	0	0	0	0.70
12. Hobbies	8.8	3.7	0.31	0.80	7.0	0	0.38	0.68
13. Spatial disorientation	42.2	7.4	0.47	0.79	14.3	10.0	0.16	0.70
14. Using household appliances	15.5	7.4	0.37	0.80	7.1	10.0	0.27	0.69

SDC: subjective decline cognitive.

The reliability, measured by Cronbach's alpha, for CFI self-report and partner report was 0.80 (95%CI 0.73–0.86) and 0.70 (95%CI 0.48–0.85), respectively. Cronbach's alpha varied less if each item was deleted in both versions (Table 2). Corrected item-total correlations ranged from 0.21 to 0.57 in CFI self-report and from 0.07 to 0.66 in CFI partner report (Table 2).

A total sample of CFI self-report score did not correlate with age, education, and gender; there was a significantly positive correlation with GAI (rS=0.413, p<0.001) and GDS (rS=0.444, p<0.001). Some questions and cognitive tests depicted a weak correlation: question 2 (questions repetition) with MMSE total score (rS=-0.251, p=0.033) and questions 4 (use of written reminders) and 12 (hobbies) with delayed recall of Logical Memory (rS=-0.252, p=0.032 and rS=-0.311, p=0.008, respectively). In addition, question 12 correlated with Category Verbal Fluency (rS=-0.246, p=0.037), delayed recall of Rey–Osterrieth Complex Figure (rS=-0.341, p=0.003), TMB (rS=0.266, p=0.024), and IQ (rS=-0.242, p=0.044).

In the SCD group, only GAI (rS=0.345, p=0.020) and GDS (rS=0.383, p=0.009) also showed significant correlations with the CFI scores. MMSE showed a significant correlation with questions 2 (rS=-0.328, p=0.028) and 4 (use of written reminders; rS=0.313, p=0.036). In the control group, education (rS=-0.403, p=0.037) and letter verbal fluency (rS=-0.385, p=0.047) correlated with CFI.

A total sample of CFI partner report did not correlate with CFI self-report (rS=0.017, p=0.936) neither with anxiety nor depression measures. Only question 13 (spatial disorientation) correlated with age (rS=0.447, p=0.029). Among the cognitive tests, MoCA had a significantly negative correlation with question 2 (rS=-0.474, p=0.019) and IQ with question 13 (rS=-0.466, p=0.022).

**Figure 2:**
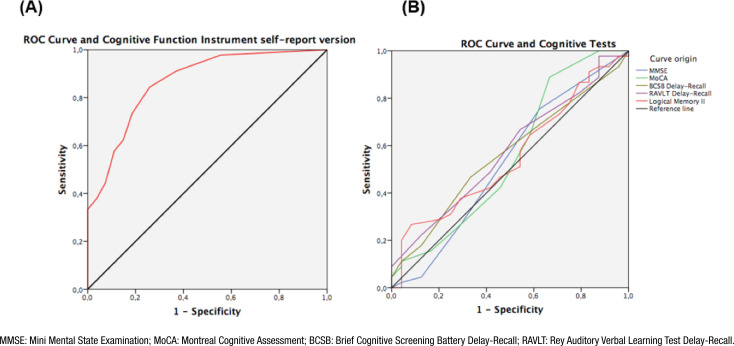
Receiver Operating Characteristic (ROC) curve was used to calculate the area under the curve (AUC) with a 95% confidence interval (95%CI) in a sample with subjective cognitive decline (SCD group) and without decline (control group). (A) Cognitive Function Instrument Receiver Operating Characteristic curve (AUC=0.865 with 95%CI 0.779–0.951, p<0.001). (B) Cognitive tests: Mini Mental State Examination (MMSE; AUC=0.529 with 95%CI 0.377–0.680, p=0.696), Montreal Cognitive Assessment (MoCA; AUC=0.556 with 95%CI 0.402–0.709, p=0.450), Brief Cognitive Screening Battery Delay-Recall (BCSB; AUC=0.562 with 95%CI 0.422–0.701, p=0.402), Rey Auditory Verbal Learning Test Delay-Recall (RAVLT; AUC=0.562 with 95%CI 0.435–0.714, p=0.310), and Logical Memory II (AUC=0.552 with 95%CI 0.411–0.693, p=0.480).

In ROC curve analysis, the AUC of the CFI self-report was 0.865 (95%CI 0.779–0.951, p<0.001) (Figure 2). A CFI self-report cutoff score of 2.0 was the one that best distinguished the SCD group from the control group, with a sensitivity of 73.3 and a specificity of 81.5%. Positive and negative predictive values were 86.8 and 65.7%, respectively. In contrast, the CFI partner report did not present a good accuracy to distinguish SCD participants from controls (AUC=0.618, 95%CI 0.392–0.844, p=0.334).

## DISCUSSION

The final version of both CFI self-report and partner report had a high level of acceptability as an assessment tool among participants with and without SCD. As expected, the individuals with cognitive complaints had higher scores in the CFI self-report than those without memory complaints. Conversely, the SCD and control groups did not show differences in neuropsychological tests, revealing the subjective nature of this syndrome, when cognitive tests are incongruent with the perception that one's memory is getting worse.

In addition to the CFI self-report score, the two groups differed in their scores on the anxiety (GAI) and depression (GDS) scales, even excluding patients diagnosed with psychiatric disorders (e.g., major depression), depressive, and anxious symptoms correlated with the CFI self-report score. One explanation is that individuals with depressive and/or anxious symptoms are more attentive and concerned about their negative perceptions, including the sensation of cognitive decline^2^. However, other hypotheses have been raised in the literature. Some authors discussed whether the first episode of a late-life depression could be a manifestation of prodromal AD, a risk factor for the development of dementia, or both^2,23^. Thus, the relationship between anxiety symptoms and SCD in older adults is still surrounded by uncertainties, whether anxiety is a consequence of perceived decline or an independent predictor for conversion to dementia^24^.

Cronbach's alpha values were substantial, indicating an acceptable internal consistency for the instrument. It mirrors the results from the original study for the CFI self-report (alpha of 0.78), but CFI partner report (alpha of 0.85)^16^. In the Italian version, the alpha was 0.77 and 0.78 retrospectively^25^, while in the Norwegian version, the values were 0.86 and 0.94, both higher than ours^26^. In turn, Li et al. calculated the interclass coefficient to assess 3-month test–retest reliability, and results were 0.76 in CFI self-report and 0.78 in CFI partner report^27^.

In addition, AUC values proved to be entirely satisfactory in the ROC curve, indicating the good accuracy of the CFI self-report to discriminate subjects with SCD from those without, better than several cognitive screening and memory tests. A score above 2.0 showed good sensitivity and high specificity for the diagnosis of SCD. Since it is a nonspecific syndrome and already denotes early cognitive decline stages, more specificity than sensitivity is sought in an SCD questionnaire. Although the original studies did not indicate a cutoff value^15,16^, in the Norwegian version, cutoff points of 4 and 6.5 were indicated to discriminate dementia from MCI and SCD using the CFI self-report and from SCD using the CFI partner report, respectively^26^.

In the present study, the most frequently reported cognitive complaints in CFI were “to need to use of written reminders” (question 4) and “trouble recalling names and words” (question 6). In line with our results, previous studies validated the MCFSI^15^ and the Italian version of the CFI^25^, and these two questions were also the most answered as yes or maybe. Interestingly, in our study, only half of the subjects with SCD answered “yes” or “maybe” to question 1 (“compared with 1 year ago, do you feel that your memory has declined substantially?”). In contrast, all the participants in the control group answered “no” to the first CFI question as expected.

Only verbal fluency test, TMTB, MMSE total scores, and delayed recall of a story (logical memory) were weakly correlated with some CFI questions but not with their total score. No neuropsychological tests correlated with CFI in our sample. This finding differed from other studies that showed a correlation between the CFI score and worse performance in objective cognitive tests^5,16,25^. In our study, questions 2 (repetition of questions) and 4 (needing notes) are the indicatives of more strictly amnestic complaints correlated with Logical Memory delayed, and with no other memory measure. Moreover, question 12 (difficulty in carrying out hobbies), which denotes more impairment of executive functions, was correlated with tests related to executive functions (verbal fluency, TMTB). Therefore, even if the total CFI score was not associated with an objective cognitive impairment, these results suggest that specific questions can be correlated with the cognitive domain to which they refer, respectively.

CFI has already been proven to be a useful tool and it has a good association with the progression of cognitive decline in nondemented older adults^16^. In a 48-month longitudinal study, Amariglio et al. showed that both CFI self-report and partner report scores were higher in the group of individuals with CDR progression. The group with progressively worse CDR score exhibited successive increment in CFI scores over the months of follow-up^16^. In another study, also with a 4-year follow-up, both CFI self-report and partner report scores at baseline were higher among those nondemented older adults (CDR: 0 or 0.5) who later evolved with cognitive decline^27^.

Regarding the CFI partner report, there was no difference between SCD and control groups in our study. This lack of difference between groups may be due to a small sample of participants with a filled partner version (only 33.3%). In contrast, this nondifference can be explained by the observation that self-perception of decline precedes that observed by the relatives and friends, differently from what occurs in patients with MCI and dementia who have a lack of awareness (anosognosia) of cognitive deficits^28^. Both the CFI self-report and partner report were associated with cognitive decline in longitudinal studies. However, the two versions showed different outcomes during the follow-up. While CFI self-report score had a higher score in baseline, CFI partner report score became higher after months of follow-up^16,27,29^. Noteworthy, the type of partner (e.g., being spouse or not) can influence the quality of information and, consequently, the prediction of cognitive decline in the long term^30^.

To the best of our knowledge, there is only one published study correlating CFI scores with AD biomarkers. In a cross-sectional survey with 4,486 pre-MCI older adults, Sperling et al. highlighted that individuals with positive amyloid PET displayed higher scores on both CFI self-report and partner report^31^. Interestingly, they showed that CFI self-report had a similar effect size between the amyloid groups and neuropsychological tests. However, further studies are needed to establish an association between the CFI scores and the diagnosis of prodromal AD using biomarkers.

The main limitation of this study is the sample size, especially the number of participants who had the CFI partner report filled out. Another limitation is the high level of education in the sample. As it is a self-administered instrument, it will be necessary to study samples with less schooling. Also, we used a single question (“*Do you feel like your memory is becoming worse?”*) as the criterion to define the SCD group. However, as there is no gold standard questionnaire for the diagnosis of SCD, we opted for a question used in other studies that showed a good predictive value for the progression to MCI or dementia^3,32,33^.

The main contribution of our study was the translation and clinical validity of an SCD instrument that has been widely used in longitudinal cohorts and studies with AD biomarkers in reference centers to be used in Brazil, since we lack questionnaires adapted to our language and culture to assess SCD. Furthermore, the CFI self-report proved to be an instrument with good accuracy and easy applicability to identify SCD among older adults.

CFI proved to be a questionnaire with good sensitivity and specificity for the diagnosis of SCD, with the greatest accuracy among many neuropsychological measures. Future longitudinal studies will be essential to assess the potential of CFI in predicting conversion to MCI or dementia in the Brazilian population. Also, studies with biomarkers should be interesting to explore the role of the CFI questionnaire in subjects with a diagnosis of prodromal AD in our country.
