# Erratum: Translation, cross-cultural adaptation, and validity of the Brazilian version of the Cognitive Function Instrument

**DOI:** 10.1590/1980-5764-DN-2021-0057-ER

**Published:** 2026-09-25

In the article "Translation, cross-cultural adaptation, and validity of the Brazilian version of the Cognitive Function Instrument", with DOI code number https://doi.org/10.1590/1980-5764-DN-2021-0057, published in the Dement Neuropsychol 2022 March;16(1):79-88

**Where it was written:**

Supplementary Tables 1 and 2 show the final version of the CFI. Eventually, a combination of the two translations was used for the synthetic version (items 1, 4, 7, 8, 9, 10, 11, and 13), while in other items, one of the translations was chosen (items 2, 3, 5, 6, 12, and 14).

**It should be:**

Supplementary Tables 1 and 2 (available at https://www.demneuropsy.org/wp-content/uploads/2022/03/DN-2021.0057-Supplementary-Material.docx) show the final version of the CFI. Eventually, a combination of the two translations was used for the synthetic version (items 1, 4, 7, 8, 9, 10, 11, and 13), while in other items, one of the translations was chosen (items 2, 3, 5, 6, 12, and 14).

Editor-in-Chief: Ricardo Nitrini. https://orcid.org/0000-0002-5721-1525

